# Visual analogue scales: scale recalibration by patients with dementia and their proxies

**DOI:** 10.1007/s11136-012-0226-3

**Published:** 2012-07-05

**Authors:** Alexander M. M. Arons, Paul F. M. Krabbe, Gert Jan van der Wilt, Marcel G. M. Olde Rikkert, Eddy M. M. Adang

**Affiliations:** 1 Department of Epidemiology, Biostatistics and HTA, Radboud University Medical Centre, P.O. Box 9101, 6500 HB Nijmegen, The Netherlands; 2 Department of Epidemiology, University Medical Center Groningen, University of Groningen, P.O. Box 30.001, 9700 RB Groningen, The Netherlands; 3 Department of Geriatrics, Alzheimer Center Nijmegen, Radboud University Medical Centre, P.O. Box 9101, 6500 HB Nijmegen, The Netherlands

**Keywords:** Scale recalibration, Health-related quality of life, Dementia, Visual analogue scale, Bias, Patient, Proxy

## Abstract

**Background:**

Visual analogue scales (VAS) are often used to measure health-related quality of life (HRQoL). However, when such scales contain ambiguous anchors like “best imaginable health state,” they produce answers that are difficult to interpret, as such anchors are interpreted differently by respondents of different age. This phenomenon that people’s interpretation of subjective response scales changes in response to changing circumstances is known as scale recalibration. The current study attempts to investigate whether scale recalibration in a patient sample with cognitive limitations and proxies differs from the general population.

**Methods:**

The participants in the current study were 151 pairs of community-dwelling patients with dementia and their proxies. They were administered three VASs with different upper anchors; (A) “best imaginable health state,” (B) “best imaginable health state for someone your age,” and (C) “best imaginable health state for a 25-year-old.” From literature, we inferred a conceptual model for the general population that predicts the ordinal relationship of the VASs to be B ≥ A ≥ C. This rank order is tested by repeated measure ANOVA’s in the aforementioned populations.

**Results:**

VAS scores of patients with dementia were in line with the conceptual model. Proxy VAS scores for assessing patient HRQoL were not in line with the model: A > B > C. In addition, proxy VAS scores for assessing their own health were not in line with the model: A > B > C.

**Conclusion:**

Patients with dementia use the VAS in a similar way to the general population. Proxies assessing either patients or themselves differ from the general population.

## Introduction

Health-related quality of life (HRQoL) is a patient-reported outcome measure that is frequently assessed in health-care evaluation research. There are two distinct ways to do this. The first uses multidimensional instruments, grounded in classical test theory that generates summary scores on several domains. One such instrument is the MOS-Short Form-36 [1]. The second uses instruments that provide an overall HRQoL value in a single metric. Known as preference-based HRQoL classification systems, these are based on specific valuation techniques. The resulting preference-based HRQoL values (variously called utilities or preference scores) are frequently used in cost-effectiveness studies.

One commonly applied HRQoL valuation technique is the visual analogue scale (VAS). It has been used for patients in numerous disease areas, but also for the general public to value specific health states [2–5]. It is generally accepted that its application is highly feasible and that it shows moderate-to-good test–retest reliability. This technique is often used to assess patients’ HRQoL in longitudinal studies [6–8]. However, it has some methodological flaws; in particular, it is prone to end-aversion and context bias. Furthermore, it is not embedded in a clear underlying theoretical measurement framework [9–12]. In addition, the anchors in these scales are potentially ambiguous, a point on which this article expands [13].

Most VASs used to measure health states adopt “perfect health” or, alternatively, “best imaginable health state” as the upper anchor. However, such notions are ambiguous. Individual respondents might understand the upper anchor differently in light of their health status. For example, a 50-year-old to whom “perfect health” means as “perfect health for someone my age” will probably give a different answer than a 50-year-old respondent to whom it means “perfect health for someone without age-related problems (for example 25 years old).” Since most studies use a VAS with an ambiguous upper anchor their results might be difficult to interpret, especially in groups with a wide range in age. Indeed, respondents might disregard the upper anchors entirely or misunderstand their intended meaning when giving HRQoL ratings if they are not provided with a well-defined frame of reference [14]. The phenomenon that people’s interpretation of subjective response scales changes in response to changing circumstances is known as scale recalibration [15–17].

Scale recalibration is one of three possible mechanisms that allow for “response shift.” This concept refers to a change in HRQoL outcomes attributable to changes in the meaning of HRQoL, as understood or experienced by a respondent. In addition to scale recalibration, a response shift can reflect a change in the relative importance to a respondent of the component domains of HRQoL (reprioritization) or a redefinition of one’s meaning of HRQoL itself (reconceptualization) [15, 18].

Scale recalibration effects have been investigated by Ubel et al. [13] in a representative sample of the elderly randomly selected from the Health and Retirement Study (HRS). Their study used VASs with three distinct upper anchors: “perfect health,” “perfect health for someone your age,” and “perfect health for a 20-year-old.” They found that the three anchors were probably understood differently, as they yielded different results. It is questionable whether these findings can be generalized to specific patient groups, however.

One of the objectives of the study on which the present work is based was to investigate whether the findings reported by Ubel et al. have generic properties. To that end, this paper reports on an exercise to replicate their findings in two specific populations. The first consists of patients with cognitive limitations, that is, dementia. This population is of particular interest because a valid and reliable measurement of HRQoL is not as straightforward in dementia as it is in many other disease areas. A decline in intellectual capacity, semantic knowledge, and episodic memory as well as deficits in judgment and insight, might affect the validity of reported HRQoL values.

The second group consists of informal caregivers (proxies) of the dementia patients. Proxy outcomes are often used to assess the patient’s HRQoL when dementia progresses to a stage in which patient assessment is no longer meaningful. One problem with using proxies for this purpose is that different cognitive processes might be at work, and these could affect the reported values. For example, proxies might prioritize domains differently than the patients or conceptualize different domains of HRQoL. Thus, when they rate patients, proxies may report different values than those drawn patient self-assessment or proxy self-assessment. In addition, proxies might “project” part of their own HRQoL problems onto the patients’ HRQoL. Of equal importance to the validity of HRQoL values of patients with cognitive limitations is the answer to whether scale recalibration occurs when assessing the HRQoL of others.

The aim of the current exercise is to investigate scale recalibration in dementia patients and proxies. It constitutes a test of whether HRQoL values elicited on distinct VASs in these specific groups are comparable to those in the general population.

## Methods

### Respondents

The current exercise draws its respondents from the AD-Euro study (a cost-effectiveness study of post-diagnosis care in dementia) [19]. The AD-Euro study sought to recruit 220 patient–proxy dyads and follow them for a 1-year period. Participants were recruited by a multidisciplinary memory clinic (MMC) physician directly after diagnosis. The inclusion criteria were as follows: patients with a newly diagnosed dementia fulfilling DSM-IV-TR criteria and having a clinical dementia rating (CDR; 0–3) score of 0.5–2: 0 for none, 0.5 for questionable/very mild, 1 for mild, 2 for moderate, and 3 for severe dementia [20, 21]. Patients were excluded if (1) their life expectancy was less than 1 year, (2) they were living in a nursing home, (3) they were already evaluated as being suitable for living in a nursing home, (4) data collection was difficult (e.g., due to severe visual/hearing/language impairment, mood disorder, or behavioral disturbances), (5) the patient’s general practitioner did not agree to participate, (6) they were already participating in another study, (7) they had visited the MMC for a second opinion, (8) the travel distance between the MMC and the patient’s residence was more than 50 km, and (9) they had a definite indication for MMC follow-up. In addition to the CDR, the Mini-Mental State Examination (MMSE) was administered, although scores on this instrument were not taken as an inclusion or exclusion criterion [22]. For more details regarding the AD-Euro study, the reader is referred to Meeuwsen et al. [19].

Measurements for the current investigation were done at 6 months. Data were collected by trained interviewers who administered the questionnaires (paper format) and the response tasks at the patient’s home. Interviews were planned in advance with both the patient and the proxy so that data on both participants could be collected on the same day and at the same location. The current exercise includes only dyads of whom patients had completed at least the VAS A (see below) rating at 6 months.

### Measures

**Fig. 1:**
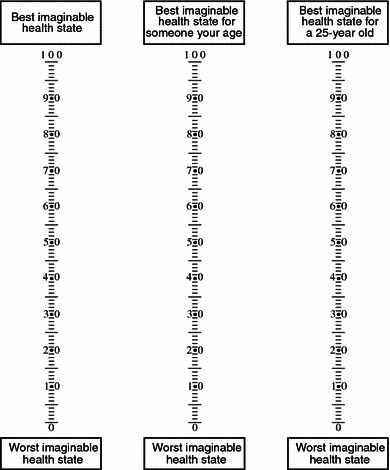
Three VASs with different upper anchors (from left to right: VAS A, VAS B, and VAS C)

All HRQoL measures were particularizations of the EQ-5D visual analogue scale (VAS A) [23]. VAS A is a 20 cm vertical “thermometer” ranging from 0 to 100 where 0 is defined as the “worst imaginable health state” and 100 is defined as “best imaginable health state” (Fig. 1). In addition to VAS A, two adapted VASs were administered to both patients and proxies. These two VASs had different upper anchors, but identical lower anchors. The first alternative (VAS B) had a score of 100, defined as “the best imaginable health state for someone your age” (for proxies reporting on patients, a score of 100 was defined as “the best imaginable health state for someone the age of the patient”). The second alternative (VAS C) had a score of 100, defined as “the best imaginable health state for a 25-year-old”. Participants indicated their HRQoL first on VAS A, then on VAS B, and finally on VAS C. There was no between-participant randomization of the VASs.

### Conceptual model

The rationale behind the model (provided below) was as follows. When people assessed their HRQoL on VAS A, it was uncertain whether or not age would be a factor in their assessment. When people assessed their HRQoL on VAS B, age should have been taken into account because it was now salient. When people assessed their HRQoL on VAS C, age should have been taken into account on a stable anchor. In this conceptual model, HRQoL values were thus composed of two factors: age and disease. Disease should be regarded as any health-related condition apart from aging that causes HRQoL to deteriorate, conditions such as rheumatoid arthritis and depression or Alzheimer’s disease. Both of these factors would influence the VAS values. Three different cases are described below to illustrate the potential scoring differences in this conceptual model (Table 1).

**Table 1:** Three different cases to illustrate the influence of age on scoring on a VAS with an ambiguous anchor

Case	Age	Decline age^a^	Decline disease	VAS A	VAS B	VAS C
1	50	10	0	90–100	100	90
2	50	10	25	65–75	75	65
3	80	22	35	43–65	65	43

^a^A decline in health for age is calculated by 0.4 points for every year above the age of 25 and is for illustrative purposes only

An underlying assumption was that respondents would assess their health in terms of decline. They would presumably start assessing their position on a scale from the position of perfect health and then they would assess which aspects are suboptimal. Furthermore, the assessments of decline were assumed to consist of two factors, namely age and disease. Thus, respondents who assessed their HRQoL would do so by identifying the decline in health caused by age separately from that caused by disease. The values on these factors were subsequently summed to capture an overall decline in health; that number would then be subtracted from the value for perfect health.

In addition, the three VASs were assumed to have identical interval properties. This implies that a respondent who identified a 20-point decline in HRQoL on a particular VAS because of disease would subtract these 20 points on each of the other VASs. Furthermore, health was assumed to decline with age; HRQoL was assumed to have a positive correlation with health, disease a negative one, and respondents were assumed capable of meaningfully assessing their decline (disutility) with regard to age and disease.

The abovementioned assumptions were rather strict; however, relaxing some or all of these assumptions would have made the conceptual model unnecessarily complicated. As this paper should be regarded as a tentative exercise to reproduce the results found by Ubel et al. in two specific populations, we chose to formulate a simple model, one that presents our thoughts in an easily interpretable way.

#### Ordinal relationship

In mathematical terms, scores on the different VASs can be expressed as follows:

1 $$ Y_{{{\text{VAS}}\,{\text{A}}}} = 100 - D_{D } - (\alpha \times D_{A} ) + \varepsilon , $$

2 $$ Y_{{{\text{VAS}}\,{\text{B}}}} = 100 - D_{D } + \varepsilon , $$

3 $$ Y_{{{\text{VAS}}\,{\text{C}}}} = 100 - D_{D } - D_{A } + \varepsilon . $$

where *Y* represents the score on the VAS, *D*_*D*_ the disutility of the health state caused by disease, *D*_*A*_ the disutility of the health state caused by age, and α a chance parameter that corrects for the potential incorporation of *D*_*A*_ as a factor. This means that, on the individual level, a respondent can either incorporate age-related disutility into the HRQoL value or not, so α takes on a value of 1 or 0. At the population level, α will represent the average of all individuals and will thus be a number between 0 and 1. The term ε represents a random measurement error component. The appendix provides a derivation for the ordinal relationship

4 $$ Y_{{{\text{VAS}}\,{\text{B}}}} \ge Y_{{{\text{VAS}}\,{\text{A}} }} \ge Y_{{{\text{VAS}}\,{\text{C}}}} . $$

### Hypotheses and analyses

The ordinal relationship presented by the above conceptual model was evaluated by testing the following two hypotheses separately for three groups (patients self-assessed, patients as assessed by proxies, and proxies self-assessed).

1. *Y*_VAS B_ is significantly larger than *Y*_VAS A_ and *Y*_VAS C_.
2. *Y*_VAS A_ is significantly smaller than *Y*_VAS B_ and significantly larger than *Y*_VAS C_.

Repeated measures ANOVAs with Bonferroni adjustments for multiple comparisons were used on the scores of the three VASs. Additionally, the agreement was examined on VAS A, VAS B, and VAS C between patient–proxy dyads (assessments on patients) by means of limits of agreement (LoA) [24]. The difference scores (patient–proxy) of each VAS were compared with the difference scores for the other VASs by means of a repeated measures ANOVA.

In order to investigate whether the type of proxy (spouse vs. child) affected VAS ratings, the next step used a MANOVA with *Y*_VAS A_, *Y*_VAS B_, and *Y*_VAS C_ as dependent variables. The patient–proxy relationship was taken as a fixed factor and patient age and proxy age were taken as covariates. The limited sample size did not permit additional analyses of informative subgroups.

## Results

### Respondents

In total, 175 patient–proxy dyads were included in the AD-Euro study at baseline. The current exercise used data collected after 6 months. In total, 151 respondents were included in the analyses (some patient–proxy dyads were not, due to attrition). The mean age of the patients was 78 (SD 5.8) years and 60 % were female. Alzheimer’s disease was the most prevalent diagnosis (62 %), followed by mixed dementia (28 %), vascular dementia (5 %), and other (4 %). Patient CDR scores were 0.5 (5.3 %), 1 (80.1 %), and 2 (14.6 %). The mean MMSE scores were 23 (SD 3.7). Patient–proxy relationships were defined as partners (56 %), children (38 %), or other (6 %). Proxies were 64 (SD 13.0) years of age and 70 % were female. The respondent characteristics for this study and Ubel et al.’s are given below for comparison (Table 2).

**Table 2:** Sample characteristics

	Patients (*n* = 151)	Proxies (*n* = 151)	Ubel VAS A (*n* = 305)	Ubel VAS B (*n* = 309)	Ubel VAS C (*n* = 309)
Age; mean (SD)	78.4 (5.8)	64.5 (13.0)	68.1 (10.1)	68.0 (10.2)	68.9 (10.0)
Gender; % female	59.6	69.5	58.8	59.3	58.4
Activities of daily living; % Reporting limitations^a,b^	39.1	21.9	20.3	21.4	24.9

^a^In our study defined as scoring “some” or “severe” problems on the domains of mobility or self care on the EQ-5D

^b^In the study by Ubel et al. defined as indicating any problems on either walking, eating, bathing, dressing, getting in/out of bed, using the toilet, or picking up a dime

### Scale recalibration

**Fig. 2:**
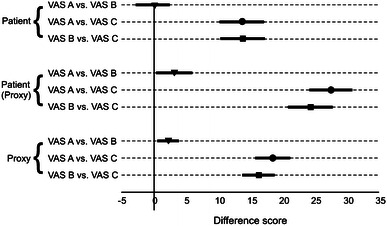
Difference scores in visual analogue scales in patients, proxies, and patients assessed by proxies

A statistically significant scale recalibration effect was seen in ratings by patients, ratings of patients by proxies, and proxies by themselves (Fig. 2). The scoring order on the different VASs by patients was VAS B = VAS A > VAS C. This is in line with the rank order as predicted by the conceptual model. Patient-by-proxy assessment produced results that did not correspond to the model’s predictions. The scoring order was VAS A > VAS B > VAS C. Proxy self-assessment showed a pattern similar to patient-by-proxy assessment. Descriptive statistics of the ratings by patients, proxies, and proxy-assessed patients on the three different VASs are provided in Table 3.

**Table 3:** Mean VAS scores of patients, proxies, and patients assessed by proxies on all 3 VASs

Type of VAS	Valid n	Mean	SD
Patient VAS A	151	73.3	13.4
Patient VAS B	137	74.0	14.5
Patient VAS C	125	59.9	18.8
Patient-by-proxy VAS A	148	67.2	14.6
Patient-by-proxy VAS B	148	63.8	18.3
Patient-by-proxy VAS C	148	39.4	19.9
Proxy VAS A	149	77.8	13.2
Proxy VAS B	149	75.7	15.0
Proxy VAS C	147	59.5	17.6

### Agreement

**Fig. 3:**
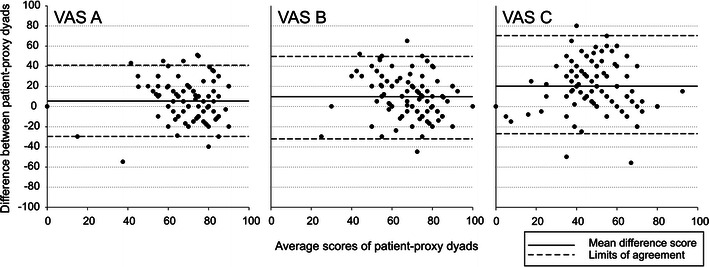
Limits of agreements between patient–proxy dyads on VAS A, VAS B, and VAS C

There was a wide range in agreement between patients and proxies on the VASs (Fig. 3). However, the spread in difference scores remains large on all three VASs, Nonetheless, there was a significant increase in differences on the agreement between the VASs (multivariate *p* < 0.001). Specifically, the difference between VAS A and VAS B increased ($\bar{x}$ = 3.39, *p* = 0.026), the difference between VAS A and VAS C increased ($\bar{x}$ = 14.52, *p* < 0.001), and the difference between VAS B and VAS C increased ($\bar{x}$ = 11.12, *p* < 0.001). The LoA of VAS A, VAS B, and VAS C were −30.13–41.38, −32.16–50.20, and −27.81–68.08, respectively.

### Subgroup analyses

Ratings on VAS A, VAS B, and VAS C were not significantly influenced by the age of the patient (*p* > 0.1) or proxy (*p* > 0.1), nor the type of patient–proxy relationship (spouse vs. child, *p* > 0.1)

## Discussion

This study has investigated whether potential scale recalibration by dementia patients and their informal caregivers (proxies) differs from findings in the general population. Three VASs with different upper anchors were used to elicit HRQoL ratings.

A comparison between a VAS with the upper anchor denoted as “best imaginable health state” (VAS_best-health_) and another VAS defining it as “best imaginable health state for someone your age” (VAS_your-age_) revealed similar results for the patients. This might suggest that age-related decline is not incorporated in patient self-rated HRQoL on the VAS_best-health_. An alternative explanation is that patients do not experience an age-related decline in health. However, when the anchors were changed to perfect health for a 25-year-old person (VAS_25-years_), they did assess their HRQoL as lower, suggesting that patients do recognize that their health has declined with age. Therefore, it seems likely that patients do not incorporate age-related decline in health in their HRQoL ratings on the VAS_best-health_. This implies that the meaning of VAS_best-health_ to patients does not differ from its meaning to the general population (as reported by Ubel et al.). That interpretation would remain in line with the conceptual model presented in this article. Despite the cognitive decline that the patient sample suffered from, it appears that VASs are understood identically by the patients and the general population. Thus, there seems to be no need for researchers and clinicians to question the interpretation of results obtained by such instruments.

Reviewing the patient-by-proxy assessments, a different pattern emerges. Proxies give the patients’ health state a higher rating on VAS_best-health_ than on VAS_your-age_. Surprisingly, this is not in line with the predictions of the conceptual model. The only explanation that would fit the model is that proxies consider age-related decline to be negative (so proxies would have judged patients to have shown improvement in their HRQoL as they aged), but this seems highly unlikely. A more plausible explanation is that the anchors mean different things to patients and proxies. One mechanism that could drive such a discrepant attribution is the decreasing scope for ambiguous responses in the mind of the respondent. Consider the following hypothetical example. It is possible that proxies understand VAS_best-health_ implicitly as “the best imaginable health state for such a patient.” When they subsequently rate the health state of the patient on VAS_your-age_, the proxies realize that, compared to people of a patient’s age, the patients are actually doing worse. Thus, they rate the patients lower on VAS_your-age_. Consider another example: a proxy might reason as follows when using VAS_best-health_ to rate a patient: “I know she has cognitive deficits, but she doesn’t seem to mind.” When that same proxy subsequently rates the same patient on VAS_your-age_, the reasoning could be that: “She might not mind her cognitive deficits, but compared to a normal person of her age her health has declined.” Another possible explanation is that proxies have less opportunity to adjust their own coping mechanisms when shifting from VAS_best-health_ to VAS_your-age_ to VAS_25-years_. Thus, as they rate the HRQoL of a patient, they are partly rating their own provision of care. Such cognitive processes might be one of the reasons there was poor agreement between patients’ self-assessments and proxies reporting on patients. In addition, they would fit the trend of decreasing agreement from VAS_best-health_ to VAS_your-age_ to VAS_25-years_.

Interestingly, when proxies assess their own HRQoL, their scores are lower on VAS_your-age_ than on to VAS_best-health_. Apparently, they are judging their HRQoL as less than normal for people of their own age. This observation contradicts the conceptual model and it differs from what Ubel et al. found, namely that people did not give different scores for VAS_best-health_ and VAS_your-age_. A possible explanation for this discrepancy is that proxies see themselves as active caregivers, which creates more stress and a greater burden than expected in a “normal” person their age. These effects could decrease HRQoL among proxies, depending on their coping style [25].

The finding that VAS_best-health_ is rated higher than VAS_your-age_ is in contrast to what Ubel et al. found, although this divergence might be explained by differences in the research designs. In the study of Ubel et al., the respondents only assessed their own HRQoL; they did not rate the HRQoL of others. Furthermore, they used a between-subject design, so that each individual received only one version of the VAS.

A limitation of the work presented here is that a within-subject design was used, without random ordering of the VASs (because this explorative exercise was not the main objective of the broader AD-Euro study). Therefore, it cannot be ascertained whether the scale recalibration effects are an artifact of order effects or whether they are genuinely present and contrary to previous findings. Nevertheless, when subjective HRQoL values are preferred and proxies are used to assess these, researchers and clinicians should be aware of the potential discrepancy between the way patients and proxies understand the scale. Given the research design underlying the current paper, it is impossible to determine whether these discrepancies are systematic or not. Further research with longitudinal and between-subject designs could be initiated to investigate and overcome this limitation.

The work reported in the present paper has another limitation. Although it is highly likely that the different ratings were induced mainly by scale recalibration, other causative factors cannot be ruled out. First of all, the formulation of the anchors might have caused not only a change in scale recalibration, but also some elements of reconceptualization or reprioritization. For example, the explicit description of age in VAS_25-years_ might have triggered recall of what was important to the respondent at that age, thereby inducing a reconceptualization or reprioritization of HRQoL. A second observation that might explain the difference in results between VAS_best-health_ and VAS_your-age_ is that a substantial proportion of the respondents gave little to no attention to the upper anchor of VAS_best-health_, since this was the first VAS they were confronted with [14]. However, this would not explain the differences found between VAS_your-age and_ VAS_25-years_, nor those between VAS_best-health_ and VAS_25-years_.

It should be noted that the assumption of equal interval levels on the different VASs is strong. Relaxing this assumption would demand additional transformations to arrive at comparable HRQoL ratings. However, in the current model, such transformations would involve undefined parameters and therefore cannot be computed directly. In theory, such transformations would allow for comparable HRQoL estimates of all three VASs. Future research should be conducted on potential additional (chance) parameters that were not included in the conceptual model presented here. That investigation could elucidate whether the model can be extended in such a way that the results of the present work would no longer be violations of the conceptual model.

The current work was performed on a sample that consisted mostly of patients with mild dementia. The choice of this population might affect the generalizability of the findings. For example, one could imagine that for proxies who take care of patients at a more severe stage of dementia, reconceptualization and reprioritization might play a bigger role than reported in this article. These reactions might then manifest themselves as different patterns of VAS ratings than those presented here. Furthermore, the current paper cannot adequately distinguish among the various cognitive processes that might be at work when proxies evaluate patient HRQoL. It is possible that assessing another person’s HRQoL in general leads to the reported patterns, though these could also be caused by the burden that the proxies have experienced. Future research should address these issues. In addition, a replication of proxy assessment on different VASs with a between-subject design is recommended. Such a study would be necessary to investigate whether the results reported here are replicable and representative of proxy assessment but also to correct for potential ordering effects.

In conclusion, it is most likely that the use of VAS_best-health_ in patients with dementia will lead to scale recalibration in patients with dementia. In addition, their understanding of VAS_best-health_ will be identical to VAS_your-age_. Researchers and clinicians need to give these effects due consideration when using VAS_best-health_ in groups ranging widely in age. Measurement by proxy does not comply with the conceptual model presented here. Therefore, future research should be done on HRQoL values assessed by proxies to identify additional parameters. Their incorporation could be a step toward explaining the deviations from the proposed model but also the discrepancy between the expected results and the patient self-assessed scores.

## Appendix

Here we will derive an ordinal scoring between *Y*_VAS *A*_, *Y*_VAS *B*_ and *Y*_VAS *C*_. Starting with *Y*_VAS *A*_ and *Y*_VAS *B*_. First, we express Eq. (2) in terms of *D*_*D*_. This gives the equation

$$ D_{D } = 100 - Y_{{{\text{VAS}}\,B}} + \varepsilon . $$

Substituting $100 - Y_{{{\text{VAS}}\,B}} + \varepsilon$ in *Y*_VAS *A*_ results in

$$ Y_{{{\text{VAS}}\,A}} = 100 - 100 + Y_{{{\text{VAS}}\,B}} - \varepsilon - \left( { \alpha D_{A } } \right) + \varepsilon \Leftrightarrow $$

$$ Y_{{{\text{VAS}}\,A}} = Y_{{{\text{VAS}}\,B}} - \left( { \alpha D_{A } } \right). $$

As α is defined as a non-negative parameter constrained between 0 and 1 and ε is a random term that disappears from the equation it follows that

$$ Y_{{{\text{VAS}}\,B}} \ge Y_{{{\text{VAS}}\,A}} . $$

Now we derive a scoring order for *Y*_VAS *C*_.

$$ D_{D } = 100 - Y_{{{\text{VAS}} \,C}} - D_{A } + \varepsilon . $$

Substituting $100 - Y_{{{\text{VAS}} \,C}} - D_{A } + \varepsilon$ in *Y*_VAS *A*_ results in

$$ Y_{{{\text{VAS}} \,A}} = 100 - 100 + Y_{{{\text{VAS}} \,C}} + D_{A } - \varepsilon - \alpha D_{A } + \varepsilon \Leftrightarrow $$

$$ Y_{{{\text{VAS}} \,A}} = Y_{{{\text{VAS}} \,C}} + (1 - \alpha )D_{A } \Leftrightarrow $$

$$ Y_{{{\text{VAS}} \,C}} = Y_{{{\text{VAS}} \,A}} - (1 - \alpha )D_{A } . $$

As α is defined as a non-negative parameter constrained between 0 and 1 and ε is a random term that disappears from the equation it follows that

$$ Y_{{{\text{VAS}} \,C}} \le Y_{{{\text{VAS}} \,A}} . $$

We suppose a transitive relation so that if

$$ Y_{{{\text{VAS}}\,B}} \ge Y_{{{\text{VAS}} \,A}} , $$

and

$$ Y_{{{\text{VAS }} \,A}} \ge Y_{{{\text{VAS}} \,C}} , $$

then

$$ Y_{{{\text{VAS}} \,B}} \ge Y_{{{\text{VAS}} \,C}} . $$

Now we arrive at the following VAS ordinal relationship:

4 $$ Y_{{{\text{VAS}} \,B}} \ge Y_{{{\text{VAS }} \,A }} \ge Y_{{{\text{VAS}} \,C}} . $$
